# Regulatory mechanism of Reelin activity: a platform for exploiting Reelin as a therapeutic agent

**DOI:** 10.3389/fnmol.2025.1546083

**Published:** 2025-01-27

**Authors:** Mitsuharu Hattori

**Affiliations:** Department of Biomedical Science, Graduate School of Pharmaceutical Sciences, Nagoya City University, Nagoya, Aichi, Japan

**Keywords:** Reelin, ApoER2, VLDLR, oligomerization, proteolysis, Alzheimer’s disease

## Abstract

Reelin is a secreted glycoprotein that was initially investigated in the field of neuronal development. However, in recent decades, its role in the adult brain has become increasingly important, and it is now clear that diminished Reelin function is involved in the pathogenesis and progression of neuropsychiatric and neurodegenerative disorders, including schizophrenia and Alzheimer’s disease (AD). Reelin activity is regulated at multiple steps, including synthesis, posttranslational modification, secretion, oligomerization, proteolytic processing, and interactions with extracellular molecules. Moreover, the differential use of two canonical receptors and the presence of non-canonical receptors and co-receptors add to the functional diversity of Reelin. In this review, I summarize recent findings on the molecular mechanisms of Reelin activity. I also discuss possible strategies to enhance Reelin’s function. A complete understanding of Reelin function and its regulatory mechanisms in the adult central nervous system could help ameliorate neuropsychiatric and neurodegenerative disorders.

## Introduction

Reelin is a secreted protein that is functionally deficient in the mutant *reeler* mouse, and for three decades, has been explored in the neuronal development field (D’Arcangelo et al., 1995; Katsuyama and Terashima, 2009; Sekine et al., 2014; Hirota and Nakajima, 2017; Kon et al., 2017; Santana and Marzolo, 2017; Jossin, 2020). More recently, Reelin has attracted increasing attention due to its roles in synaptic plasticity and associations with neuropsychiatric and neurodegenerative disorders, as summarized in previous reviews (Folsom and Fatemi, 2013; Guidotti et al., 2016; Ishii et al., 2016; Lane-Donovan and Herz, 2017; Scala et al., 2022; Alexander et al., 2023; Reive et al., 2024). Importantly, almost all the literature suggests that Reelin activity loss or reduction correlates with neurological symptom onset and/or exacerbation, whereas increased levels and/or activity exert protective or therapeutic effects. This is true for schizophrenia (Grayson et al., 2005; Teixeira et al., 2011; Rogers et al., 2013; Iafrati et al., 2014; Sakai et al., 2016; Sobue et al., 2018; Ibi et al., 2020; Sawahata et al., 2020; Liao et al., 2022), Alzheimer’s disease (AD) (Durakoglugil et al., 2009; Kocherhans et al., 2010; Pujadas et al., 2014; Lane-Donovan et al., 2015; Yamakage et al., 2019b; Rossi et al., 2020; Lopera et al., 2023), and autism spectrum disorders (ASD) (Lammert et al., 2017; Zhubi et al., 2017; Sánchez-Sánchez et al., 2018; Morrill et al., 2022). Thus, many neuropsychiatric and neurodegenerative disorders could be ameliorated by enhancing Reelin function in the adult brain. However, this does not hold true for non-neuronal inflammatory or cardiovascular diseases (Ding et al., 2016; Gowert et al., 2017; Calvier et al., 2021, 2023), where reduced or lost Reelin function may be advantageous. Given the evidence showing the detrimental effects of reduced Reelin activity in different disorders, increasing Reelin function in the brain could help mitigate these conditions. This review summarizes recent findings and provides insights on the molecular mechanisms regulating Reelin function, and examines how to increase Reelin function in the adult central nervous system.

In this review, apolipoprotein E receptor 2 (ApoER2, also known as Lrp8) and very low-density lipoprotein receptor (VLDLR) are referred to as canonical Reelin receptors. These molecules are unequivocally essential for most Reelin functions, from both biochemical and genetic perspectives (D’Arcangelo et al., 1999; Hiesberger et al., 1999; Trommsdorff et al., 1999; Dlugosz and Nimpf, 2018). Several other molecules have been reported to bind to Reelin, and are referred to as non-canonical receptors or co-receptors, depending on the proposed function or experimental results as I will discuss later. The intracellular signaling cascades modulated by Reelin include numerous other molecules, which are outlined elsewhere (Lee and D’Arcangelo, 2016; Bock and May, 2016; Hirota and Nakajima, 2017; Santana and Marzolo, 2017; Jossin, 2020) and will not be discussed here. A widely accepted key mechanism is that Reelin interacts with canonical receptors on neuronal cells and induces the tyrosine phosphorylation of an intracellular adaptor protein called Dab1 (Hiesberger et al., 1999; Howell et al., 1999, 2000; Feng and Cooper, 2009), which is then rapidly degraded (Rice et al., 1998; Howell et al., 1999; Feng et al., 2007). For simplicity, this review assumes that Reelin biological activity is indicated by increased Dab1 phosphorylation or decreased total Dab1 levels. However, it is important to note that these indicators do not encompass all of Reelin’s functional capacity as many other important events are influenced by Reelin.

## The Reelin protein structure

**Figure 1:**
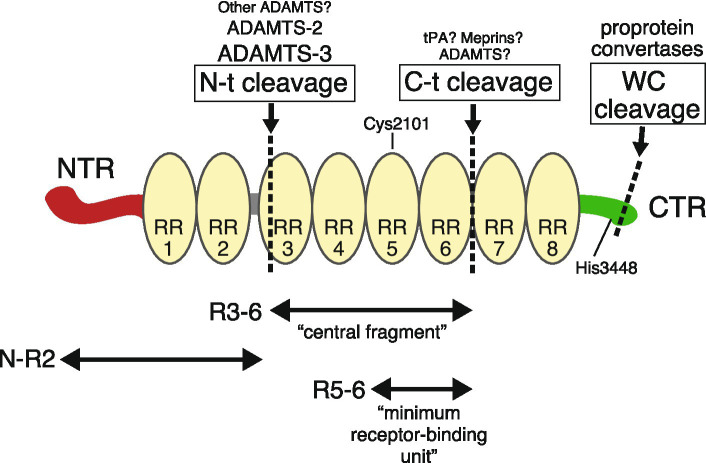
The Reelin structure and its fragments. Reelin consists of an N-terminal region (NTR, red), eight tandem Reelin repeats (RR, yellow), and a C-terminal region (CTR, green). Specific cleavage sites are shown by dotted lines. Above the name of each cleavage site are the name of the proteases responsible for it. Larger names indicate greater contribution, and those marked with a question mark indicate insufficient genetic evidence. Cys2101 in RR5 forms an intermolecular disulfide bond for dimer formation. When His3448 is substituted to Arg, Reelin activity is augmented.

In mice and humans, Reelin contains 3,461 and 3,460 amino acid residues, respectively (D’Arcangelo et al., 1995; DeSilva et al., 1997). It is one of the largest single polypeptides in mammalian species, with a molecular mass (by sodium dodecyl sulfate-polyacrylamide gel electrophoresis under reducing conditions) of 400–450 kDa. Unlike other secreted molecules implicated in neuronal function, such as netrins, semaphorins, Slits, and Wnts, Reelin or Reelin-like proteins are not found in invertebrates, but they do exist in fish. Reelin has no family relatives (there is no Reelin-2), and its overall structure is similar to no other protein. At the N-terminal end, a signal peptide directs protein secretion from cells (D’Arcangelo et al., 1995). Mature Reelin contains an N-terminal region (NTR), eight Reelin repeats (RRs) that contain an epidermal growth factor (EGF)-like domain, and finally a C-terminal region (CTR) (Figure 1). Because the 190 most N-terminal residues are weakly (25%) homologous to an N-terminal section of the extracellular matrix protein F-spondin, and the EGF-like domain often occurs in adhesion molecules, it was initially suggested that Reelin was an extracellular protein mediating neuronal adhesion and migration (D’Arcangelo et al., 1995). Although Reelin is occasionally called an extracellular matrix protein, there is no evidence to suggest that Reelin is an extracellular matrix component. Part of the NTR has sequence similarity to the RR subrepeat domain (Ichihara et al., 2001; Nagae et al., 2021). The crystal structure of recombinant Reelin mutant protein consisting of the NTR and the first RR (RR1) indicated that this region had a branched Y-shape and that two incomplete subrepeats formed one entire subrepeat structure (Nagae et al., 2021). The deletion of a part of NTR reduced Reelin’s ability to induce Dab1 phosphorylation (Kubo et al., 2002) and NTR binds to integrin (Schmid et al., 2005), suggesting that the NTR was involved in biological roles. This will be discussed later. Reelin contains two major specific sites (N-t and C-t) for proteolytic processing, from which five fragments are generated (Lambert de Rouvroit et al., 1999b; Jossin et al., 2004; Hattori and Kohno, 2021) (Figure 1). There is an additional processing site within the CTR (WC), which occurs at six residues from the C-terminus (Kohno et al., 2015). The mechanisms and significance of these fragments and associated processing will be discussed later.

Each RR is 350–390 amino acids long. Exact RR boundaries are not strictly defined, and researchers use either the original structure published by D’Arcangelo et al. (1995) or the more recent one published by Ichihara et al. (2001). The crystal structure of the third RR (RR3) shows a beta-jelly-roll fold with similarity to carbohydrate-binding domains, with the two subdomains making direct contact (Nogi et al., 2006). The central region (RR3–RR6) binds to the canonical receptors ApoER2 and VLDLR (Jossin et al., 2004). Electron micrographs of this fragment have indicated a rod-like shaped structure (Nogi et al., 2006). Yasui et al. (2007) showed that the minimum binding unit for the canonical receptor consisted of RR5 and RR6 fragments, the crystal structure of which indicated zinc (Zn^2+^) ion and critical Lys residues.

The Reelin CTR is approximately 30 residues long and is highly positively charged (D’Arcangelo et al., 1995). The primary CTR sequence is extremely well conserved across vertebrates (Nakano et al., 2007). The crystal structure of RR8 and CTR indicated that RR8 shows similarities to the other RR structures and that the CTR is flexible and works as a structurally distinct domain (Turk et al., 2022). The first proposed CTR function showed that it was essential for Reelin secretion, which stemmed from two observations. One indicated that a mutant protein expressed in *reeler* mice with the Orleans mutation (*Reln^rl-Orl^*) lacking a part of RR8 and CTR was not secreted (de Bergeyck et al., 1997). The other indicated that an artificially truncated Reelin protein lacking a part of RR8 and the CTR was not secreted (D’Arcangelo et al., 1997). However, it was later observed that Reelin without the CTR was in fact secreted (Lambert de Rouvroit et al., 1999a; Nakano et al., 2007). Also, the reason why mutant Reelin proteins were not secreted was not because they lacked a CTR, but because Reelin misfolded if any RR was abruptly terminated (Nakano et al., 2007). The reactivity of an anti-RR8 antibody was influenced by the CTR, suggesting that this somehow affected the RR8 structure (Kohno T. et al., 2009). In silico, biochemical, and physicochemical analyses indicated that CTR has an *α*-helical structure with a flexible region and binds to sulfated glycosaminoglycans (Lopera et al., 2023). CTR functions are further discussed below. Possibly due to its large size, the existence of cleaved and degradation products and complex glycosylation processes, the entire three-dimensional Reelin structure has not been reported.

## Regulation of Reelin expression at transcriptional and translational levels

The molecular mechanisms regulating Reelin expression are largely unknown both in embryonic and postnatal brains. In the forebrain during embryonic and early postnatal periods, Reelin is secreted mainly from Cajal–Retzius (CR) cells (Ogawa et al., 1995; Nakajima et al., 1997; Schiffmann et al., 1997; Alcántara et al., 1998). In the postnatal brain, Reelin is mainly secreted from interneurons (Pesold et al., 1998; Rodriguez et al., 2000; Vílchez-Acosta et al., 2022) and by projection neurons in layer II of the entorhinal cortex (Chin et al., 2007; Stranahan et al., 2011; Kobro-Flatmoen et al., 2016). A recent study using single molecule fluorescent *in situ* hybridization has revealed that various kinds of glutamatergic and GABAergic neurons express Reelin in the postnatal prefrontal cortex (Silva-Hurtado et al., 2024). Reelin is secreted from neuronal precursors in the nuclear transitory zone and from postmitotic granular neurons, respectively (D’Arcangelo et al., 1995; Ogawa et al., 1995; Pesold et al., 1998). In the striatum, Reelin is expressed in the GABAergic medium spiny neurons (Pardo et al., 2023).

Studies on the mechanism regulating Reelin expression were initially conducted in CR cells. The first such study was by Ringstedt et al. (1998) who showed that brain-derived neurotrophic factor (BDNF) suppressed Reelin expression in CR cells. As this study also reported morphological and other changes in CR cells, it appeared likely that BDNF altered CR cell properties rather than specifically altering Reelin gene expression.

Human Reelin (*RELN*) mRNA and protein levels are downregulated in patients with schizophrenia (Guidotti et al., 2016) and ASD (Folsom and Fatemi, 2013). *RELN* mRNA is reduced in the postmortem frontal cortex in patients with ASD, and concomitantly, methyl-CpG binding protein 2 and DNA methyltransferase 1 binding to the *RELN* promoter region is increased, suggesting attenuated *RELN* mRNA expression levels via this binding (Zhubi et al., 2017). In the cerebral cortex of presenilin-1 (PS1) conditional knock-out (KO) adult mice, Reelin mRNA and protein levels were significantly increased while the number of Reelin-positive cells were not changed, suggesting that PS1 decreased Reelin expression (Balmaceda et al., 2014). An ApoER2 fragment generated by *γ*-secretase (which requires PS1) was shown to influence Reelin expression in SH-SY5Y cells (Balmaceda et al., 2014). An interesting aspect of this study was that Reelin expression in the whole brain increased, even though PS1 was deficient only in excitatory neurons. Because Reelin was mainly expressed in inhibitory rather than excitatory neurons in the adult brain, the underlying mechanism remains to be investigated. In mouse embryonic fibroblasts, Ras signaling via phosphoinositide 3-kinase negatively regulated Reelin transcription (Castellano et al., 2016), but it is not clear if this also applied to neuronal cells. Recently, in postnatal cerebral cortical neurons, increased electrical activity was shown to upregulate Reelin transcription via the BDNF/TrkB pathway, but interestingly, secreted Reelin protein levels remained unchanged (Engeroff et al., 2023). It was also shown that silencing the neuronal network promoted Reelin translation without affecting transcription or secretion, suggesting that neuronal activity, in a complicated manner, controlled Reelin synthesis, whereas its secretion appeared constitutive (Engeroff et al., 2023).

## Regulation of Reelin via posttranslational modification

There is limited evidence on the posttranslational modification of Reelin, but like many other secreted proteins, it is glycosylated (D’Arcangelo et al., 1997; Botella-López et al., 2006). Investigations using lectins reported that Reelin glycosylation patterns differed between plasma and cerebrospinal fluid, suggesting that secreting cells were different (Botella-López et al., 2006). Interestingly, the fraction of Reelin that did not bind to Concanavalin A was decreased in the frontal cortex of AD patients, while amyloid *β* (Aβ) altered Reelin glycosylation in SH-SY5Y cells (Botella-López et al., 2010). How glycosylation affects Reelin function remains unknown. Other than glycosylation, there is little evidence to suggest that Reelin undergoes posttranslational modification. Reelin binds to Zn^2+^ ions (Yasui et al., 2007) but the significance of this is unknown.

## Regulation of Reelin secretion

An important question is whether Reelin secretion is regulated or not. If Reelin is involved in the regulation of neuronal plasticity in the adult brain, its secretion must be tightly regulated, presumably via neuronal activity. However, to date, studies examining the regulatory mechanisms underlying Reelin secretion are scarce. Reelin secretion from cultured cerebellar granule neurons was shown to be independent of neuronal activity and likely occurred via a constitutive secretory pathway (Lacor et al., 2000). Blocking the neuronal activity by chronic application of tetrodotoxin did not affect Reelin secretion from cultured hippocampal neurons (Groc et al., 2007). Reelin secretion from transfected hippocampal neurons was unaffected by neuronal activity, while BDNF secretion was augmented by neuronal excitation (Nakao et al., 2022). Where in the neuronal cells Reelin secretion occurs is also a question, and immunohistochemical analyses suggested that Reelin is secreted from axons (Derer et al., 2001; Martínez-Cerdeño et al., 2003). However, there is little evidence to suggest that Reelin is secreted from axon terminals like neurotransmitters. Recently, it was reported that some Reelin mutants found in patients with pachygyria inhibited the secretion of co-expressed wild-type (normal) Reelin in a dominant-negative fashion in transfected cultured cells (Riva et al., 2024). It remains to be investigated how these mutations impair the secretion.

## Reelin dimerization is required for biological activity

The most important functional event in the Reelin signaling pathway is Dab1 tyrosyl phosphorylation (Hiesberger et al., 1999; Howell et al., 1999, 2000; Feng and Cooper, 2009). How Reelin induces this modification in neurons is not fully understood, but the most current model suggests the following. Reelin is a dimer or oligomer, and by binding to canonical receptors, Reelin induces their dimerization, oligomerization, or clustering. Then Dab1, bound to the cytoplasmic domain of canonical receptors, forms a dimer or oligomer. Dimeric or clustered Dab1 is then recognized by Src family tyrosine kinases (SFKs) and undergoes phosphorylation, which activates SFKs, and this positive feedback loop induces more robust Dab1 phosphorylation. This model explains most of the data, e.g., Dab1 dimerization is sufficient for its phosphorylation (Strasser et al., 2004; Wang and Cooper, 2017), while higher-order Dab1 and canonical receptor clustering may lead to more robust Dab1 phosphorylation (Dlugosz et al., 2019). However, three questions remain regarding this model. Firstly, is oligomeric Reelin the only active endogenous ligand, or does dimeric Reelin have sufficient function? Secondly, does Reelin form oligomers before secretion or does this occur in the extracellular space? Thirdly, is Reelin dimerization and/or oligomerization regulated? In this section, I will discuss Reelin dimerization.

Reelin forms a dimer (Kubo et al., 2002) via a disulfide bond between Cys2101 in RR5 (Yasui et al., 2011). A Reelin mutant where Cys2101 was replaced with Ala (C2101A) did not form a dimer, but assembled into a non-covalent oligomer with a slightly reduced average size (Yasui et al., 2011). The C2101A mutant bound to the extracellular domain of ApoER2 and VLDLR with slightly lower affinity than wild-type Reelin, but did not induce Dab1 phosphorylation in cultured cerebral cortical neurons (Yasui et al., 2011). The dimeric form of a Reelin recombinant protein consisting of R3–6 bound to ApoER2 and VLDLR ectodomains with a higher affinity than its monomeric form (Turk et al., 2021). The monomeric R3–6 protein did not seem to signal even at a high concentration when assayed for phosphorylation of ribosomal protein S6 as an indicator in cultured cerebral cortical neurons (Turk et al., 2021). These studies indicated that Reelin dimerization was a prerequisite step for biological activity. It should be emphasized that the full-length Reelin C2101A mutant still formed oligomers, but they were not biologically active (Yasui et al., 2011). This observation suggests two important points: first, there is another self-association site(s) in full-length Reelin, and second, Reelin oligomerization is not sufficient for signaling activity, suggesting that a specific type of oligomerization or a higher order structure is required. To the best of my knowledge, there is no evidence suggesting that dimerization via Cys2101 is regulated.

## Regulation of Reelin activity via oligomerization

I now speculate on how Reelin oligomerizes and to what extent oligomerization is required for full Reelin activity. Before discussing these issues, I introduce an important antibody in Reelin research; the anti-Reelin monoclonal antibody CR-50.

The CR-50 monoclonal antibody was established by immunizing the brain lysate of wild-type mice to *reeler* mice (Ogawa et al., 1995). CR-50 was first reported in the same year as the Reelin gene was identified (Ogawa et al., 1995). CR-50 recognizes the Reelin NTR (D’Arcangelo et al., 1997; Nagae et al., 2021) and inhibits Reelin function *ex vivo* (Miyata et al., 1997; Zhao et al., 2004; Cariboni et al., 2005; Sinagra et al., 2005; Matsuzaki et al., 2007; Durakoglugil et al., 2009; Zhou et al., 2016; Dairaghi et al., 2018; Orcinha et al., 2021) and *in vivo* (Nakajima et al., 1997; Heinrich et al., 2006; Antonioli-Santos et al., 2017). CR-50 (at very high concentrations) blocks interactions between Reelin and 293 T cells expressing VLDLR (D’Arcangelo et al., 1999). Whether or not CR-50 attenuates Reelin’s binding to ApoER2 or to the plasma membrane of cultured neurons has not been reported. When compared to another frequently used anti-NTR monoclinal antibody G10, CR-50 bound more favorably to oligomeric than dimeric Reelin, while it did not bind to monomeric, reduced, or denatured Reelin (Ishii et al., 2019). Importantly, the recombinant NTR protein, like full-length Reelin, tended to oligomerize, with CR-50 inhibiting this oligomerization (Utsunomiya-Tate et al., 2000). Mutant Reelin lacking part of the NTR, which included the CR-50 epitope, did not form oligomers and showed a much weaker ability to induce Dab1 phosphorylation (Utsunomiya-Tate et al., 2000; Kubo et al., 2002). Together with data showing that canonical receptor (Strasser et al., 2004; Dlugosz et al., 2019) or Dab1 (Wang and Cooper, 2017) cluster formation was required to induce Dab1 phosphorylation, the following scenario was postulated; Reelin is synthesized and secreted as a dimer from Reelin-expressing neurons. Probably after secretion, Reelin forms oligomers, which can be inhibited by CR-50. Oligomeric Reelin binds to canonical receptors and induces their cluster formation. Dab1, bound to intracellular regions in clustered canonical receptors, also forms clusters, which induce SFK activation and/or Dab1 phosphorylation. This model seems to successfully explain many results and observations.

But another question remains; is Reelin oligomerization absolutely necessary to induce Dab1 phosphorylation or is it just a booster? Unfortunately, it remains unclear if CR-50 abrogates recombinant Reelin’s ability to induce Dab1 phosphorylation in cultured cerebral cortical neurons. However, several groups tested whether the NTR-less Reelin fragment could induce Dab1 phosphorylation in cultured cerebral cortical neurons. The first study was conducted by Jossin et al. (2004) who showed that the R3–6 fragment (Ile1220-Ser2664, 0.2–0.3 nM) had comparable activity as full-length Reelin. Nogi et al. (2006) showed that the R3–6 fragment (Ser1222-Ile2661) at 1 nM and 10 nM weakly and robustly, respectively, induced Dab1 phosphorylation in cultured cerebral cortical neurons, which suggested that this fragment was not as potent as full-length Reelin. Our group reported that an alkaline-phosphatase (AP) fusion of the central fragment (AP-R3–6, Thr1235-Gly2663) at 0.5 nM induced Dab1 phosphorylation in cultured cortical neurons, but this activity was weaker than full-length Reelin by approximately 3-fold (Uchida et al., 2009). AP is a dimer, which could be good or bad for central fragment function. Lee et al. (2014) reported that the R3–6 fragment (the exact region was not provided, at >5 nM) induced Dab1 phosphorylation in cultured cerebral cortical neurons, but was approximately 5-fold weaker than full-length Reelin. Yasui et al. (2007) reported that the R5–6 fragment was a minimum receptor-binding unit and its monomeric form induced Dab1 phosphorylation in cortical neurons at 30 nM, while in the oligomerized state, this occurred at 3 nM. Finally, Dlugosz et al. (2019) showed the R3–6 fragment (Leu1221-Ile2661, 30 nM) did not induce canonical receptor clustering in overexpressed cultured cells but still affected certain downstream signaling processes.

Despite research group and methodological differences, it appears that Reelin (and its central fragment) can activate downstream signaling without oligomerization, and the oligomerization enhances activity by at least several fold. However, if CR-50 inhibits Reelin function *in vivo* by inhibiting Reelin oligomerization, it follows that Reelin oligomerization occurs only after its secretion. Furthermore, if oligomerization in the extracellular milieu is an endogenous Reelin mechanism, it remains unclear how it is regulated. Further analyses are required to fully understand the regulatory mechanisms regulating Reelin’s actions in the extracellular space and to exploit them for therapeutic purposes.

## Regulation of Reelin activity via proteolysis

The proteolytic processing of extracellular signaling proteins can serve multiple roles, including converting inactive propeptides into active forms, modulating different functions, release from the cell-surface matrix, and inactivation. Lambert de Rouvroit et al. (1999b) conducted pioneering research on Reelin proteolysis, and were the first to demonstrate that the Reelin protein in the mouse brain consists of multiple cleaved fragments generated by metalloproteinases. As mutant Reelin in *Reln^rl-Orl^* mice is not secreted (de Bergeyck et al., 1997) and does not produce cleaved fragments (Lambert de Rouvroit et al., 1999b), it was strongly suggested that proteolytic cleavage occurred in the extracellular space after secretion. They also identified two main cleavage sites N-t and C-t (Lambert de Rouvroit et al., 1999b; Jossin et al., 2004) and estimated that the former lay between RR2 and RR3 (Jossin et al., 2003). It was later found that N-t cleavage occurrs between Pro1244 and Ala1245 (Koie et al., 2014), which fell within RR3 either by the original proposal of RRs (D’Arcangelo et al., 1995) in which RR3 starts with Leu1231, or by the newly proposed one (Ichihara et al., 2001) in which RR3 starts with Thr1235. Therefore, the “genuine” central fragment generated by N-t and C-t cleavage was 10–25 residues shorter than the recombinant central fragment used by us and other groups (Jossin et al., 2004; Nogi et al., 2006; Yasui et al., 2007; Uchida et al., 2009; Dlugosz et al., 2019).

In terms of the physiological significance of Reelin cleavage, Jossin et al. (2004) proposed that it was the liberation of the active central fragment. We partially purified the protease that cleaved the N-t site from the culture supernatant of cerebral cortical neurons and used it to compare activity between full-length Reelin and its completely cleaved products (e.g., a mixture of N-R2 and R3-8C fragments) (Kohno S. et al., 2009). We observed that the cleaved fraction had a much weaker activity for the induction of Dab1 phosphorylation (Kohno S. et al., 2009). From these results, together with NTR-mediated oligomerization findings described in the previous sections, the most plausible mechanism was that the central fragment could induce Dab1 phosphorylation, but its activity was weaker than full-length Reelin. However, these observations were generated *in vitro* using primary cultured neurons under artificial conditions, thus, there is no guarantee that they fully mimicked *in vivo* Reelin function. To address this, it was necessary to identify the protease(s) responsible for Reelin proteolysis.

Regarding the protease(s) mediating N-t cleavage, a few years after Reelin was identified, Goffinet’s group tested several protease inhibitors in embryonic brain explants and suggested that proteolysis was mediated by “adamalysin” type metalloproteases, later called ADAM (a disintegrin and metalloproteinase) or ADAMTS (ADAM with a thrombospondin type 1 motif) family metalloproteases (Lambert de Rouvroit et al., 1999b). It was then reported that ADAMTS-4 (Hisanaga et al., 2012; Krstic et al., 2012) and ADAMTS-5 (Krstic et al., 2012) mediated Reelin cleavage at both N-t and C-t sites *in vitro.* However, quantitative analysis indicated that ADAMTS-4 was not a Reelin-cleaving enzyme secreted by cerebral cortical neurons (Hisanaga et al., 2012). We then showed that ADAMTS-3 was an endogenous enzyme that mediated N-t cleavage (Ogino et al., 2017). ADAMTS-3 is expressed in and secreted from cerebral cortical neurons; it is matured by the proprotein convertase family, binds to heparin, exclusively cleaves N-t but not C-t sites, and most importantly, culture supernatants from cerebral cortical neurons of ADAMTS-3 KO mice cannot cleave N-t site (Ogino et al., 2017). In the embryonic and early postnatal cerebral cortex and hippocampus of ADAMTS-3 KO mice, the amount of N-R2 fragment (i.e., the product of N-t cleavage) was greatly decreased but not completely eliminated, indicating that there is an additional enzyme(s) mediating N-t cleavage (Ogino et al., 2017). ADAMTS-2 was suggested as a possible candidate (Yamakage et al., 2019a; Hattori and Kohno, 2021). Dab1 levels were decreased in the cerebral cortex in embryonic and early postnatal ADAMTS-3 KO mice, strongly suggesting that ADAMTS-3 inactivated Reelin (Ogino et al., 2017). To prove this, we established cleavage-resistant Reelin knock-in (KI) mice by introducing a mutation into the N-t site (Okugawa et al., 2020). N-R2 fragment and Dab1 levels were markedly decreased in the cerebral cortex and hippocampus of these mice, indicating that Reelin activity increased by the loss of N-t cleavage. Moreover, the hippocampal layer was disturbed in the KI mice, but this phenotype was ameliorated by the deficiency of one copy of the Reelin gene. Thus, the hippocampal abnormality was attributed to excess Reelin activity due to lost N-t cleavage (Okugawa et al., 2020). In sum, biochemical and genetic evidence indicate that in mouse cerebral cortex and hippocampus, N-t cleavage is mediated mainly by ADAMTS-3 and this is the major inactivation process of full-length Reelin. To the best of my knowledge, there has been no genetical or *in vivo* observation that supports the idea that N-t cleavage liberates active Reelin fragments.

Much less is known about C-t cleavage. As described, ADAMTS-4 and ADAMTS-5 were shown to cleave C-t sites, but there is no evidence to support their contribution to Reelin cleavage *in vivo*. Tissue plasminogen activator (tPA) is a serine protease that is highly expressed in the hippocampus and cleaves Reelin at C-t sites *in vitro* (Krstic et al., 2012; Trotter et al., 2014), but Reelin and fragment levels were unchanged in tPA KO mouse brains (Trotter et al., 2014). C-t cleavage mediated by culture supernatants from cerebellar granular neurons occurred between Ala2688 and Asp2689 (Sato et al., 2015). Meprins are an astacin family of metalloproteases that also cleave C-t sites *in vitro;* however, *in vivo* evidence is lacking (Sato et al., 2015). Since C-t cleavage separates the receptor binding unit (R5–6) from CTR, a co-receptor binding unit (see below), it is presumed to reduce (but not completely) the activity of Reelin. However, no studies ever have validated this.

There is also a third cleavage site in CTR (Kohno et al., 2015). CTR contains four consecutive Arg residues, which is the recognition site for proteases of proprotein convertase family. This site, which we named the WC cleavage site, occurs between Arg3455 and Ser3456 and liberates a six amino acid peptide (Kohno et al., 2015). In cultured cells, WC cleavage occurred in secretory pathways, and thus Reelin in culture supernatants did not contain the most C-terminal six residues (Kohno et al., 2015). Reelin with the last six residues (i.e., full-length protein) bound more strongly to cultured cerebral cortical neuron surfaces when compared to Reelin missing these residues (i.e., Reelin used in practically all *in vitro* studies) (Kohno et al., 2015). Detailed WC cleavage mechanisms require further study.

**Figure 2:**
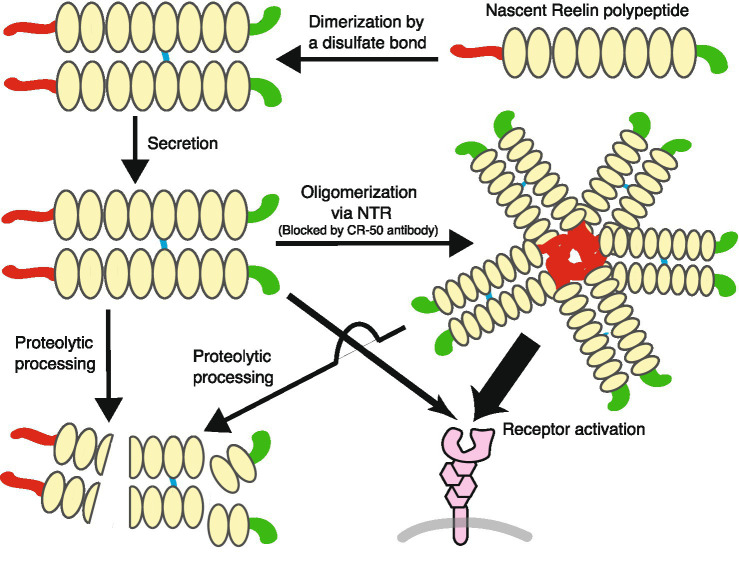
The life of Reelin protein. Reelin forms a dimer by a disulfide bond before secretion. Secreted dimeric Reelin protein oligomerizes, but the extent of this oligomerization is not clear: the cartoon is depicted as a pentamer, but this is merely a model, not a claim that such a thing exists. The oligomerized Reelin is probably more active than the Reelin dimer (indicated by the thickness of the arrows). Reelin protein in the extracellular space is eventually processed by proteases.

As the summary of the last four sections, the life of Reelin protein is schematically shown in Figure 2.

## Regulation of Reelin functions via its canonical receptors

ApoER2 and VLDLR were first suggested as Reelin receptors because double-KO mice lacking both showed brain phenotypes similar to *reeler* and *Dab1*-deficient mice (Trommsdorff et al., 1999). It was later shown that both receptors directly bound to Reelin (D’Arcangelo et al., 1999; Hiesberger et al., 1999). Although dissociation constants between Reelin and these receptors varied between studies, it is consistent that Reelin binds to ApoER2 several to 10 times more strongly than VLDLR (Andersen et al., 2003; Yasui et al., 2011; Turk et al., 2021). In the embryonic cerebral wall, ApoER2 was identified as the major Reelin-binding protein while less so for VLDLR, as visualized by staining wild-type, ApoER2-KO, and VLDLR-KO mice using AP-fusion probe of the central fragment (Uchida et al., 2009). Single KO mice for *ApoER2* and *VLDLR* showed different brain structure phenotypes, which were largely explained by their expression patterns (Hack et al., 2007). Basically, ApoER2 was essential for late-generated neocortical neuronal migration, whereas VLDLR primarily acted as a stop signal for migrating neurons, preventing their invasion into marginal zones (Hirota and Nakajima, 2020).

Some important differences between ApoER2 and VLDLR are evident in terms of the intracellular events elicited by Reelin. ApoER2 uniquely recruits JNK-interacting proteins, which are not associated with VLDLR (Stockinger et al., 2000). ApoER2 and VLDLR are sorted into different membrane domains: ApoER2 is associated with raft domains and cause specific receptor fragment production, while VLDLR is found in non-raft domains and directs Reelin for degradation (Duit et al., 2010). Cultured HEK293 cells expressing ApoER2 produced filopodia/lamellipodia and their sizes were increased by Reelin treatment, whereas VLDLR-expressing cells decreased in size (Dlugosz et al., 2019). These differences, in addition to distinct expression patterns and affinity to Reelin, are thought to contribute to fine-tune Reelin functions.

## Regulation of Reelin functions via non-canonical receptors and binding molecules

Apart from ApoER2 and VLDLR, several other Reelin receptors, which are termed non-canonical receptors in this review, have been proposed. There have also been a few “co-receptor” candidates for the canonical receptors. The former includes EphB receptor tyrosine kinases, integrin, and amyloid precursor protein (APP), while the latter includes ephrin-B and neuropilin-1 (Nrp1).

Reelin binds to the extracellular domain of EphB receptor tyrosine kinases and induces their activation (Bouché et al., 2013). However, this did not cause Dab1 phosphorylation, and Reelin-induced Dab1 phosphorylation was not affected in neurons lacking EphB receptor tyrosine kinases (Bouché et al., 2013). Double-KO mice deficient for *EphB1* and *EphB2* exhibited CA3 hippocampal pyramidal neuron positioning defects that were similar to *reeler* mice, suggesting that EphB receptor tyrosine kinases and canonical Reelin receptors were required for hippocampal development elicited by Reelin (Bouché et al., 2013). EphB receptor tyrosine kinases were shown to regulate synapse formation, spine development, and central nervous system plasticity (Henderson and Dalva, 2018). However, it remains unclear if Reelin modulates these events via these kinases.

It was reported that Reelin directly interacted via its NTR with the integrin α3β1 extracellular domain (Dulabon et al., 2000; Schmid et al., 2005). Cortical neuron positioning was abnormal in integrin α3-KO mice, but phenotypes were not similar to *reeler* mice (Schmid et al., 2004). Integrin β1 inactivation specifically in migrating neurons generated no developmental defects (Belvindrah et al., 2007). Therefore, the importance of the interaction between Reelin and integrins was not supported by genetic evidence so far.

As a putative noncanonical receptor for Reelin, APP has generated the great interest (Pohlkamp et al., 2017). APP is a transmembrane protein that is cleaved to produce Aβ, a toxic peptide that aggregates to form amyloid plaques in patients with AD. As APP and canonical Reelin receptors share some characteristics (i.e., the extracellular domain binds to ApoE, the intracellular domain binds to Dab1, and they undergo intramembrane cleavage via *γ*-secretase), Reelin and APP interactions have been extensively investigated. Hoe et al. (2006) showed that Reelin increased interactions between APP and Dab1 in primary cultured cortical neurons and that the central region of Reelin bound to the APP E1 domain (Hoe et al., 2009). Rice et al. (2013) systematically re-examined interactions between candidate APP ligands, and interestingly, were unable to reproduce many published results, but did show that Reelin bound to APP and thereby inhibited its ectodomain shedding. Dab1 hypomorphic mouse phenotypes were exacerbated and ameliorated by the overexpression of pathogenic APP mutant and the deficiency of endogenous APP, respectively (Pramatarova et al., 2008). Therefore, the genetic interaction between APP and Dab1 is obvious. However, it remains unclear if these effects are mediated by Reelin-APP interactions. An alternative hypothesis is that APP functions downstream of Reelin, and not as its receptor. For example, Reelin treatment increased Dab1 phosphorylation and decreased associations between APP and Dab1, which did not occur in *Fyn* KO neurons (Minami et al., 2011), suggesting that Reelin modulated APP localization in a canonical receptor-dependent manner.

Finally, Lee et al. (2014) found that Reelin can induce the phosphorylation of extracellular signal-regulated kinase (ERK) 1/2 in cultured cerebral cortical neurons and that this effect was blocked in the presence of the lipoprotein receptor antagonist, receptor-associated protein (RAP). Furthermore, the central fragment of Reelin did not induce Erk1/2 phosphorylation, and Dab1-deficiency in the neurons only partially decreased Erk1/2 phosphorylation (Lee et al., 2014). These observations led them to hypothesize the involvement of an unidentified Reelin receptor that triggers a signal transduction pathway that leads to Erk1/2 activation (Lee et al., 2014).

In Zebrafish tectum, Reelin was gradually distributed probably by binding to heparan sulfate proteoglycans (Di Donato et al., 2018). Mouse Reelin also bound heparin via its NTR (Tan et al., 2008) and CTR (Lopera et al., 2023). These observations suggest that like many other extracellular diffusible ligands, Reelin may be modulated by sulfated proteoglycans in mammalian brain, but so far, the evidence is inadequate.

## Regulation of Reelin functions by co-receptors

**Figure 3:**
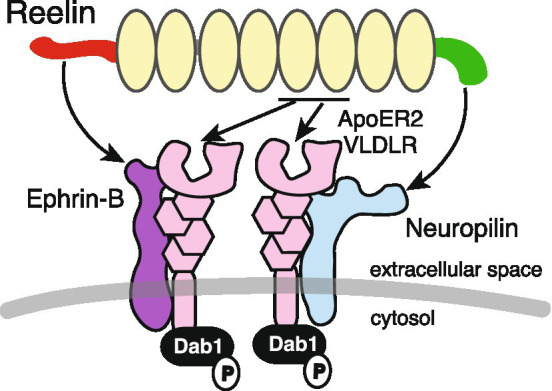
Reelin, canonical receptors, and co-receptors. ApoER2 and VLDLR canonical receptors bind to Reelin at repeats 5 and 6. Ephrin-B and Nrp1 bind to the Reelin NTR and CTR, respectively, and form complexes with canonical receptors. Dab1 is associated with the cytosolic domain of canonical receptors and is phosphorylated when Reelin binds to these receptors.

As canonical receptors do not possess enzymatic activity in their cytoplasmic domains, the presence of signaling co-receptors has been hypothesized (Bock and May, 2016). Currently, two molecules have been proposed as co-receptors for canonical receptors: ephrin-B and Nrp1 (Figure 3).

Sentürk et al. (2011) proposed that ephrin-B family proteins, transmembrane ligands of Eph receptor tyrosine kinases, bound to Reelin and functioned as a component of the canonical receptor signaling. Ephrin-B3 was shown to form a complex with ApoER2 and VLDLR in the embryonic mouse brain (Sentürk et al., 2011). Ephrin-B clustering increased Dab1 phosphorylation levels at Tyr232 (which is not commonly assessed by most researchers), and Reelin-induced Dab1 phosphorylation was attenuated in cultured cerebral cortical neurons from ephrin-B2/ephrin-B3 double-KO mice (Sentürk et al., 2011). However, these observations were overshadowed by image duplication issues (Erratum in Nature, 478, 274 (2011)), and key results were challenged by several independent groups (Pohlkamp et al., 2016).

Reelin without CTR (Reelin-ΔC) was less potent than the full-length CTR-bearing Reelin (Reelin-FL) in inducing Dab1 phosphorylation (Nakano et al., 2007). CTR is encoded by a single exon and Reelin-ΔC is produced by alternative polyadenylation (Lambert de Rouvroit et al., 1999a). The interaction between cultured cerebral cortical neurons and Reelin-ΔC was weaker than that for Reelin-FL, suggesting that the Reelin CTR bound neuronal molecules on the plasma membrane (Nakano et al., 2007). In KI mice in which the CTR was deleted, versatile neurological symptoms were identified, such as narrower marginal zones in the cerebral cortex (Kohno et al., 2015), some misplaced cerebellar Purkinje cells (Nakamura et al., 2016), abnormal hippocampal layer formation (Ishii et al., 2023), and neuropsychiatric-disease-like behaviors (Sakai et al., 2016). The same results were essentially observed in CTR-lacking mice generated by forward genetic screening (Ha et al., 2015, 2017). These observations indicated that some, but not all, Reelin functions were dependent on CTR, and it was likely that there is a co-receptor bound to Reelin-FL, but not Reelin-ΔC. The CTR is highly basic and contains four consecutive Arg residues, which are known consensus sequences for Nrp1 interactions (Teesalu et al., 2009). Nrp1 is a surface receptor with pleiotropic functions, and its ligands include class 3 semaphorins, vascular endothelial growth factor, and SARS-CoV-2 (Guo and Vander Kooi, 2015; Cantuti-Castelvetri et al., 2020). Indeed, Reelin-FL, but not Reelin-ΔC, bound to Nrp1 expressed in cultured cells (Kohno et al., 2020). Nrp1 formed a complex with the VLDLR, and the interaction between Reelin-FL and VLDLR was augmented by Nrp1 (Kohno et al., 2020). Dab1 levels were increased in cultured cerebral cortical neurons when Nrp1 was knocked down, while *in utero,* Nrp1 knock-down affected apical dendrite development and superficial neuron positioning in the developing mouse cerebral cortex (Kohno et al., 2020), consistent with *VLDLR* KO mice (Hack et al., 2007). These findings strongly supported the notion that Nrp1 was a co-receptor that functioned with canonical receptors and mediated Reelin-FL activity.

Recently, Reelin CTR has drawn considerable attention in AD research. In 2023, Lopera et al. (2023) reported that in a dominantly inherited AD family, a male member did not develop cognitive impairment until his late 60’s. He was shown to have a *PS1-E280A* mutation, which would normally have led to cognitive impairment by his late 40’s. Nonetheless, he showed no AD symptoms, even though severe amyloid plaque accumulation was evident in his brain (Lopera et al., 2023). Genome sequencing showed that he had a *RELN* mutation that substituted His3447 (His3448 in mice) to Arg (Lopera et al., 2023). KI mice with this mutation (H3448R) were generated and showed that Dab1 phosphorylation was only augmented in male brains (Lopera et al., 2023). Sex differences in the expression level and the effect of Reelin have been observed both in human and rodents (e.g., Shifman et al., 2008; van den Buuse et al., 2012; Hill et al., 2013; Sánchez-Lafuente et al., 2022; Sánchez-Lafuente et al., 2024). Thus, a point mutation in the Reelin CTR enhanced its function in a sex-dependent manner, thereby preventing cognitive abnormalities caused by amyloid accumulation in the brain. It remains unclear how this one amino acid substitution generated such a powerful mutant status and why the effects were only manifested in males.

## Concluding remarks and problems to be solved

**Figure 4:**
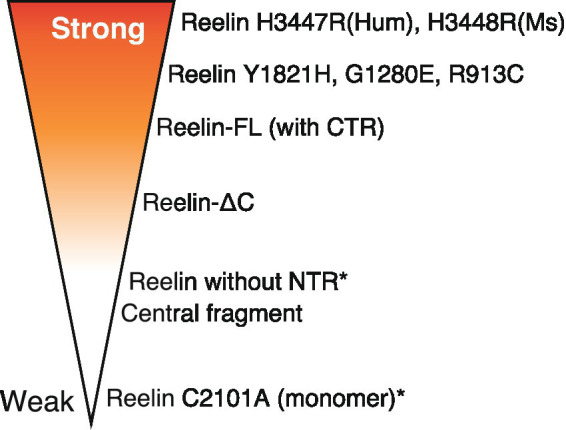
Schematic showing the biological activity (strength) of different Reelin proteins. More intense red refers to strong activity while no shading indicates the opposite. Asterisks indicate artificial proteins. The variant distribution presented in the figure is not representative of the activity magnitude.

In recent decades, considerable research efforts have successfully characterized signal transduction mechanisms underlying Reelin function. The main advancements in the last 15 years includes the clarification of the roles of each domain of Reelin, the identification of Reelin-cleaving proteases, and the identification of non-canonical receptors and co-receptors. The discovery of gain-of-function Reelin point mutation (H3447R) is a recent surprise. Recently, it was reported that point mutations in human pachygyria patients (Reelin Y1821H, G1280E, and R913C) showed stronger activity than wild-type Reelin when overexpressed in embryonic mouse brains (Riva et al., 2024). Reasons for this remain also unclear. A schematic showing the biological activity of different Reelin proteins (some artificial and some naturally occurring) is shown (Figure 4). Some aspects regarding Reelin regulatory mechanisms remain unclear and controversial. In particular, how important is oligomerization for endogenous Reelin activity? What is the significance of Reelin cleavage in the adult brain? How important are Reelin-binding molecules other than canonical receptors? The current consensus suggests that Reelin diffuses into the extracellular space and reaches target neurons or synapses, but Reelin behaviors after secretion, including oligomerization, are unclear. In embryonic and early postnatal mouse brains, ADAMTS-2 and -3 are major players implicated in Reelin inactivation, but whether this is true for human adult brains is unknown.

How can we use the knowledge to help relieve the suffering of human patients? Upregulation of Reelin transcription would be one possible way. For this purpose, we need to understand much more the transcriptional regulation of Reelin in adult brain. Upregulation of Reelin secretion does not seem like a practical approach, because there is no evidence that Reelin secretion is regulated (Lacor et al., 2000; Nakao et al., 2022). Perhaps the most promising approach is the inhibition of proteases that degrade and inactivate Reelin. Protease inhibitors have already been effectively utilized for diabetes (Deacon, 2020) and hypercholesterolemia (Sabatine, 2019). While developing compounds capable of crossing the blood–brain barrier is significant challenges, there are some recent advancements in technologies that make it increasingly feasible (Banks et al., 2024; Carneiro and Schaffer, 2024). Before pursuing this strategy, it is essential to determine the extent to which Reelin proteolysis contributes to the dysfunction of the adult human brain. Finally, further investigation of the gain-of-function Reelin mutations may open a new avenue for the treatment of neuropsychiatric and neurodegenerative disorders. There was a time when it appeared we almost had a comprehensive understanding of Reelin; however, this is far from the truth, and new realm of Reelin biology lies before us.
